# Long term therapeutic effects of icariin‐loaded PLGA microspheres in an experimental model of optic nerve ischemia via modulation of CEBP‐β/G‐CSF/noncanonical NF‐κB axis

**DOI:** 10.1002/btm2.10289

**Published:** 2022-01-07

**Authors:** Tushar Dnyaneshwar Desai, Yao‐Tseng Wen, Jayasimha Rayalu Daddam, Felice Cheng, Chia‐Ching Chen, Chien‐Lin Pan, Keh‐Liang Lin, Rong‐Kung Tsai

**Affiliations:** ^1^ Institute of Eye Research Hualien Tzu Chi Hospital, Buddhist Tzu Chi Medical Foundation Hualien Taiwan; ^2^ Department of Animal Science Agriculture Research Organization, Volcani Center Rishon LeTsiyon Israel; ^3^ Drug Delivery Technology Department Industrial Technology Research Institute Hsinchu Taiwan; ^4^ Department of Medical Laboratory and Biotechnology Chung Shan Medical University Taichung Taiwan; ^5^ Department of Medicine Mackay Medical College Taiwan; ^6^ Institute of Medical Sciences Tzu Chi University Hualien Taiwan; ^7^ Doctoral Degree Program in Translational Medicine Tzu Chi University and Academia Sinica Hualien Taiwan

**Keywords:** anterior ischemic optic neuropathy, granulocyte colony‐stimulating factor, neuro‐protection, noncanonical NF‐kB, PLGA‐icariin

## Abstract

An ischemic insult at optic nerve (ON) is followed by detrimental neuroinflammation that results in progressive and long‐lasting retinal ganglion cell (RGC) death and vision loss. Icariin was reported to be a safe and effective natural anti‐inflammatory drug. Herein, we evaluated the long‐term therapeutic effects of a single intravitreal injection of poly(lactide‐co‐glycolide) PLGA–icariin in a rat model of anterior ischemic optic neuropathy (rAION). Treatment with PLGA microspheres of icariin preserved the visual function and RGC density for 1 month in the rAION model. In addition, ON edema and macrophage infiltration were inhibited by treating PLGA microspheres of icariin. We found that the binding complex of icariin and CCAAT enhancer binding protein beta (CEBP‐β) significantly induced endogenous granulocyte colony‐stimulating factor (G‐CSF) expression to activate noncanonical nuclear factor kappa B (NF‐κB) signaling pathway by promoting an alternative phosphorylation reaction of IKK‐β. Activation of noncanonical NF‐κB signaling pathway promoted the M2 microglia/macrophage polarization and AKT1 activation, which prevented neuroinflammation and RGC apoptosis after ON infarct. This study concluded that protective mechanism of icariin is a CEBP‐β/G‐CSF axis‐induced noncanonical NF‐κB activation, which provides the long‐term neuroprotective effects via anti‐inflammatory and antiapoptotic actions after ON ischemia.

## 1 INTRODUCTION

Nonarteritic anterior ischemic optic neuropathy (NAION) causes a sudden, acute, and irreversible loss of vision resulting from restricted blood flow in the anterior portion of the optic nerve (ON), which leads to swelling and atrophy of the optic disc.^1^ This progresses to acute inflammation and edema, with the ultimate death of the retinal ganglion cells (RGCs) and loss of vision.^2^ Macrophage infiltration and the breakdown of the blood–optic nerve barrier (BONB) are crucial events that contribute to inflammation after ON infarct.^3^ Macrophage infiltration typically occurs within 3 days after ON infarct and resolves in about 14 days along with ON edema, contributing to the acute inflammation.^2^ This acute neuroinflammatory response catapults the disease progression leading to the loss of the RGCs. This progressive RGC loss has two major apoptotic peaks at 7–15 days and 18–21 days after ON infarct.^4^ Therefore, the prolonged period of RGC death after axonal ischemia implies that the alternative therapy for ON stroke has to consider the way to keep drug concentrations and drug efficacy for 3–5 weeks after a single dose

To address NAION, therapeutic strategies have to focus on the regulation of neuroinflammation, which includes the inhibition of macrophage infiltration, manipulation of macrophage/microglia polarization, and reduction in the expression of proinflammatory cytokines.^5^ Icariin is a flavonoid in traditional Chinese medicine that is generally used for the treatment of erectile dysfunction.^6^ It is studied for the treatment of numerous neurodegenerative diseases owing to its anti‐inflammatory potential exerted by targeting the nuclear factor kappa B (NF‐κB) signaling pathway.^7^, ^8^, ^9^ Therefore, we considered icariin is a potential drug against the neuroinflammation after ON stroke. In addition, the regulation of Nf‐κB activation by icariin remains little known in neuroinflammatory disorders. In this study, the icariin‐modulated NF‐κB activation was investigated in the experimental model of ischemic optic neuropathy.

A long‐lasting anti‐inflammatory strategy would be beneficial for countering acute inflammation and prolonged progression of the disease. One recent study reported that transgenic inhibition of astroglial NF‐κB protects from ON damage and RGC loss in experimental optic neuritis.^10^ It indicated that long‐term inhibition of NF‐κB provides a beneficial outcome for optic neuritis treatment. However, icariin has a half‐life of 0.16 ± 0.05 h in rat serum^11^, ^12^ and a half‐life of 0.06 ± 0.01 day in rat vitreous humor (Table S1). This short period of half‐life is difficult to deal with the prolonged progression of ON stroke. A poly(lactide‐co‐glycolide) (PLGA) formulation is a useful strategy for maintenance of drug concentrations more than 1 month after a single intravitreal administration in many studies.^13^, ^14^, ^15^, ^16^, ^17^ In this study, the pharmacokinetic profiles of icariin‐loaded PLGA microspheres demonstrated a long‐term sustained release in vitreous humor (Table S1). Therefore, we hypothesized that the encapsulation of icariin in PLGA allows for providing long‐term sustained release, thereby increasing half‐life of icariin to provide its therapeutic benefits throughout the slow, prolonged, and progressive ischemic optic neuropathy.

NF‐κB (p65) is an important transcriptional factor that regulates interleukin‐1β (IL‐1β) as well as many inflammatory cytokines.^18^ Its upregulation is associated with the progression of many inflammatory diseases.^19^ In an ON injury model, the NF‐κB was activated after ON transection, which may mediate RGC apoptosis.^20^ Besides, the activation of NF‐κB in the astroglia was reported in the model of optic neuritis.^10^ It has been suggested that NF‐κB activation may be a key pathological factor involved in neuroinflammatory processes secondary to RGC damage. The NF‐κB pathway assumes canonical activation in response to lipopolysaccharide or ischemic insult or any other insult, promoting inflammation or noncanonical activation, which is anti‐inflammatory.^21^ The canonical NF‐κB pathway depends on the IKK‐β phosphorylation of p65 followed by its nuclear translocation, whereas the noncanonical NF‐κB pathway is driven by the IKK‐α phosphorylation of p52 followed by its nuclear translocation. The noncanonical NF‐κB pathway mediates the regulation of immunity and the anti‐inflammatory response.^22^

We hypothesize that a single intravitreal injection of PLGA–icariin could regulate NF‐κB activation to provide neuroprotective effects in the experimental ON ischemia. To support this hypothesis, we report the findings of optical coherence tomography (OCT) imaging, retrograde fluorogold labeling, and terminal deoxynucleotidyl transferase dUTP nick end labeling (TUNEL) assay, all of which demonstrate that the pathophysiological symptoms of rat model of anterior ischemic optic neuropathy (rAION) were reduced after treatment with PLGA–icariin. Functionally, the measurements of the amplitude of the flash visual evoked potential (fVEP) indicate restoration of vision after PLGA–icariin treatment. The mechanism of icariin‐induced NF‐κB activation was further investigated by using simulation analysis, kinase assay, and immunoblotting assay in this study.

## 2 MATERIAL AND METHODS

### 2.1 Study design

In this study, rats were allocated into the healthy group, the phosphate‐buffered saline (PBS)‐treated group, the PLGA–icariin‐treated group, the free icariin‐treated group, and the placebo‐PLGA microsphere‐treated group. The rats in the healthy group were not received the operation of the rAION induction but the rats in other group were received the operation of the rAION induction. The rats in these five groups were accessed the visual function at Day 28 postinfarct by using fVEP measurements. Moreover, the protection of RGC from death and macrophage infiltration were evaluated at Day 28 postinfarct in the healthy group, the PBS‐treated group, and PLGA–icariin‐treated group by using retrograde fluorogold labeling of RGCs, TUNEL assay, and ED1 staining. The ON edema was evaluated by using OCT analysis at designed time points. The signaling pathway was evaluated at Day 3 postinfract in the healthy group, the PBS‐treated group, and PLGA–icariin‐treated group by using immunoblot analysis.

### 2.2 Animals

A total of 62 outbred adult male Wistar rats weighing 150–180 g (7–8 weeks old) were utilized for this study. The rats were obtained from a breeding colony (BioLASCO Co.). They were maintained in filter‐top holding cages and fed ad libitum in controlled environmental conditions (temperature 23°C and 55% humidity) with a 12‐h light–dark cycle. Animal care and experimental procedures were performed in accordance with the ARVO Statement for the Use of Animals in Ophthalmic and Vision Research. In addition, the Institutional Animal Care and Use Committee at Tzu Chi Medical Center approved all animal experiments. All animal procedures were performed on rats anesthetized by intramuscular injection of ketamine (100 mg/kg) and xylazine (10 mg/kg) cocktail. In all experiments, we utilized Alcaine eye drops (Alcon) for local anesthesia, Mydrin‐P (Santen Pharmaceutical) for pupil dilation, and Tobradex (Alcon) for wound healing.

### 2.3 rAION induction

After anesthesia, Alcaine and Mydrin‐P were applied to the eyes of the rats. Using a 30‐gage needle, 2.5 mM of Rose Bengal (RB) in PBS (1 ml/kg animal weight) was injected intravenously through the tail vein. Immediately after the administration of RB to the rats in the PBS‐treated group, the right optic disk was exposed to an argon green laser, with a wavelength of 532 nm, size of 500 mm, and power of 80 mW (MC‐500 Multicolor Laser, Nidek Co., LTD). The rats received 12 pulses, each with a 1‐s duration.^5^ A brilliant golden glow at the ONH after RB activation by the argon laser was considered as evidence of the successful rAION induction. For the rats in the healthy group, the right optic disk was exposed to the argon laser without intravenous injection of RB. Tobradex ointment was applied, after which the rats were kept on a heating pad at 37°C until recovery.

### 2.4 Preparation of PLGA–icariin formulation

Icariin (purity ≥94%) and polyvinyl alcohol (PVA) 4‐88 were purchased from Sigma‐Aldrich. Resomer RG 502 (50‐50; MW 10,000–20,000) PLGA was purchased from Evonik Industries. Ethyl acetate (EA) was obtained from Avantor Performace Materials, LLC. Dimethylformamide (DMF) was procured from Echo Chemical Co., Ltd. Distilled water (UNISS) was used for the preparation of formulation.

Briefly, icariin and PLGA powder were dissolved in DMF and EA cosolvent and then added to 1% PVA solution, using the homogenizer at 16,000 rpm for 7 min. Afterwards, the mixture was poured quickly into another 1% PVA solution and stirred overnight at 500 rpm to completely solidify the microparticles and remove solvents. The microparticles were isolated by centrifugation at 12,000 rpm for 30 min, and washed two times with distilled water. Finally, the microparticles were lyophilized and stored at 4°C. Drug content in the icariin PLGA formulation was analyzed by using high‐performance liquid chromatography. Sample particle size was determined by Multisizer 3 Coulter Counter.

### 2.5 PLGA–icariin administration

PLGA–icariin was prepared by dissolving PLGA–icariin powder (100 mg/ml) in water for injection. PLGA–icariin was administered via a single‐dose intravitreal injection shortly after rAION induction. The intravitreal injection was performed by using a 10 μl of Hamilton syringe with a 30 G needle. The eye was treated with Alcaine drops for local anesthesia. After the general anesthesia, we selected a spot on sclera, devoid of blood vessels and slowly injected 5 μl of drug into the vitreous cavity. Tobradex eye ointment was applied to prevent infection after injection.

Based on Lipinskis rule, icariin may be difficult to pass the blood–retina barrier (Table S3) and hence the intravitreal injection may confine its therapeutic benefits to the eye system also reducing systemic side‐effects.

### 2.6 Intraocular pressure measurement

All intraocular pressures (IOPs) were measured using a rebound tonometer (TonoLab). All measurements were recorded 30 min and 1 h after intravitreal injection. The IOP was measured three times per eye, and the average value was calculated for each eye. No significant increase of IOP was found in the free icariin‐treated, placebo‐PLGA microsphere‐treated, and PLGA–icariin‐treated rats compared to that of the normal rat eye (Figure S4).

### 2.7 Optical coherence tomography

We obtained OCT measurements to measure the opening of Bruch's membrane, as it corresponds to the width of the ONH. We used a Phoenix Micron IV microscope, along with image‐guided OCT, with a longitudinal resolution of 1.8 μm and a transverse resolution of 3 μm with a 3.2‐mm field of view and 1.2‐mm imaging depth at the retina. After anesthesia, the rats were placed at an angle to the lens. Bruch's membrane opening width (ONW) was obtained via linear scanning around the optic disk. Images of the ONW were measured using the built‐in software Insight (Phoenix Research Labs). This software measures the width of the opening of Bruch's membrane on the micron scale. OCT imaging was conducted for all groups on Days 1, 2, 3, 7, 14, 21, and 28.

### 2.8 Retrograde labeling of RGCs by fluorogold

We performed fluorogold retrograde labeling of the RGC 1 week before the animals were sacrificed. The rats were anesthetized, and the skin over the skull was opened. Using the stereotaxic apparatus (Stoelting), sagittal coordinates were perforated (anterior–posterior [AP]: −6 mm, medial–lateral [ML]: −1.5 mm, dorsal–ventral [DV]: 4 mm) using a motorized dental drill. Using a Hamilton syringe, 2 μl of 5% fluorogold was injected in the superior colliculus (DV, 4 mm). The same procedure was repeated in the other hemisphere.^5^ Seven days after the fluorogold administration, the rats were euthanized, and the eyeballs were fixed in 10% formalin. The fixed retina was flat mounted on a slide. The retinas were examined by using a 400 Epi‐fluorescence microscope (Axioskop; Carl Zeiss Meditec Inc.) equipped with a filter set (excitation filter 350–400 nm; barrier filter 515 nm), as well as a digital camera (AxioCam MRm; Carl Zeiss Meditec Inc.,) and the AxioVision 4.0 software. We examined the retinas for RGCs at a distance between 1 and 3 mm from the ONH to determine the central RGC densities. At least four randomly selected areas of 62,500 μm^2^ each in the central (40% of the central area) regions of each retina were counted, and their corresponding averages were obtained as the mean density of RGCs per retina. The ImageMaster 2D Platinum 7 software (GE Healthcare) was used to quantify the number of RGC in each image.

### 2.9 Flash visual evoked potentials

At 28 days after rAION induction, fVEP was measured by opening the skin over the skull to expose the sagittal coordinates and implanting three electrodes at the primary visual cortex region of both hemispheres using stereotaxic coordinates (AP, ML, and DV; AP: −8 mm, ML: −3.0 mm). We fixed one electrode at the frontal cortex (AP: 3 mm) and measured the fVEP using the Insight, Phoenix Research Labs software. The electrode at the primary visual cortex was considered as the active (positive) electrode, the electrode at the frontal cortex was considered as the reference (negative) electrode, and the ground electrode was connected to the rat tail. We measured the fVEPs using no background Illumination, a flash intensity of 30 cd·s/m^2^, and a single flash with a flash rate of 1.02 Hz. We plotted the average of 64 sweeps on a graph. The fVEP measurements were used to calculate the amplitude from the crest of the first positive wavelet (P1) to the trough of the second negative wavelet (N2).^5^

### 2.10 Sample preparation for immunohistochemistry and TUNEL assay

Four weeks after infarct, the rats were euthanized, and their eyes were collected. The eyes were detached along with the ON and transferred to 4% paraformaldehyde to fix the protein sample for 1 day. The samples were then transferred into 30% sucrose and stored at −40°C until they settled to the bottom of the tube as a result of dehydration. The samples were then embedded in OCT molds and stored at −20°C before sectioning. When sectioning, the samples were placed in a cryostat chamber maintained at −20°C, and 30‐μm‐thick sections of the retina and ON were obtained. The sections were made such that it lies in the central region of the eye ball so as to include the attached ON. We obtained three samples on each slide.

### 2.11 Immunohistochemistry

The residual OCT compound was washed off by dipping the microscopic slides in PBS for 5 min. The boundary was traced along the sample edge using a liquid blocker to retain the reagents used during the procedure. The tissues were then blocked with 3% bovine serum albumin (BSA) in PBST at room temperature for 1 h. Then, we prepared the primary antibody in 1:100 concentrations in 3% PBST. The tissue samples were then incubated with primary antibody overnight and were subsequently washed with 1X PBS three times for 5 min each. The corresponding secondary antibody was then prepared as a 1:500 concentration in 3% PBST. The tissue samples were incubated with the secondary antibody for 1 h at 37°C in a humid chamber. Then, the samples were washed three times with 1X PBS for 5 min each. Next, the tissue samples were counterstained with DAPI along with the mounting solution using Fluoroshield™ with DAPI. The slides were stored at 4°C until image acquisition using the corresponding filters in the fluorescent microscope. The slides were kept moist throughout the procedure using wet tissue papers.

### 2.12 TUNEL assay

We performed a TUNEL assay to determine whether PLGA–icariin had the potential to rescue the RGCs from apoptosis. To ensure that equivalent fields were used for comparison, all frozen retinal sections were prepared using the samples cut at 1–2 mm distant from the ONH. The retinal cross‐sections containing microscope slides were washed three times with 1X PBS for 5 min each. The borders were then marked along the edge of the sample using a liquid blocker to restrain the reagents used during the procedure. We treated 100 μl of Proteinase K solution (20 μg/ml) on the retina cross‐sections for 45 min. The samples were then incubated with 100 μl of kit provided equilibrium buffer for 10 min at room temperature. Then, 50 μl of TdT reaction mix was prepared per the Promega, DeadEnd™ Fluorometric TUNEL System Kit. The samples were incubated with this TdT reaction mix for 1 h at 37°C in a humid chamber. Humidity was maintained by adding wet tissue papers to the chamber. The samples were then washed with PBS three times for 5 min each, followed by counterstaining with DAPI and sample mounting using Fluoroshield with DAPI. The samples were then kept at 4°C until data acquisition. Imaging was acquired from central retina to midperipheral retina by using a confocal microscope. We manually counted the signal from the TUNEL‐positive cells in the RGC layer by randomly selecting 10 high‐power fields (×200) and calculating the average.

### 2.13 Sample collection for Immunoblotting

The samples were collected at Day 3 after infarct after posteuthanasia. The eyeballs with attached ON were surgically scoped out of the rat body. The entire ON was then severed and transferred into an Eppendorf tube at −80°C. The whole‐cell protein sample was isolated using RIPA buffer, whereas the cytoplasmic and nuclear protein was isolated using the Biovision Nuclear/Cytosol Fractionation Kit following the standard protocol.

### 2.14 Western blot

Proteins were run on 4%–10% precast gradient gel obtained from Invitrogen in 1X NuPAGE MOPS running buffer. The 10–20 μg protein sample was loaded in triplicate along with 5 μl of BL Ultra prestained ladder (GeneTex). The proteins were then transferred from the gel onto a PVDF membrane using the iBlot2 system of dry transfer. For this, we used the preprepared transfer apparatus from Invitrogen. The membranes were then blocked in 5% nonfat milk in 1X TBST. The primary antibodies were prepared in 5% BSA in 1X TBST. The membrane was incubated in this ECL complex (Immobilon Western Chemiluminescent HRP substrate) complex for 3 min and then mounted on the stage of the iBright fl1000 Imaging system. The results were obtained and quantified using the iBright Analysis software (Invitrogen).

### 2.15 Kinase assay

The Promega Kinase Assay Kit was used along with PTEN pure protein. The IKK‐β from the kit was incubated with PTEN pure protein following the kit's protocol. The IKKtide from the kit was incubated with IKK‐β, which served as the positive control. Conversely, IKK‐β was incubated with the diluent containing adenosine triphosphate alone as a negative control. These three reactions were carried out in individual wells of a 96‐well plate.

The reactions were incubated for 30 min, and the luminescence was recorded using iBright FL1000.

### 2.16 In silico docking analysis

Genetic optimization of ligand docking (GOLD) a genetic algorithm (GA)‐based software, mainly utilizes an evolutionary strategy involving three genetic operators; crossovers, mutations, and migrations. GOLD imports the partial flexibility to proteins and full flexibility to inhibitors. The Icariin molecule is docked into the active site of CCAAT enhancer binding protein beta (CEBP‐β) from *Homo sapiens* and the interaction of Icariin with the active site residues are thoroughly studied using calculations of molecular mechanics. The parameters used for GA were population size (100), selection pressure (1.1), number of operations (10,000), number of island (1), and niche size. Operator parameters for crossover, mutation, and migration were set to 100, 100, and 10, respectively. Default cut‐off values are, 3.0 Å (dH‐X) for hydrogen bonds and 6.0 Å for van der Waals were employed. The default algorithm speed was selected and the inhibitor binding sites in CEBP‐β were defined within a 10 Å radius with the centroid. The number of poses for Icariin was set to 100 and early termination was allowed if the top three bound conformations of inhibitor were within 1.5 Å RMSD. After docking, the individual binding poses of Icariin was observed and the interaction with the CEBP‐β was studied. The best and most energetically favorable conformation of icariin was selected.

### 2.17 GOLD score fitness function

The four components viz, protein–ligand hydrogen bond energy (external H‐bond); protein–ligand van der Waals energy (external vdw); ligand internal van der Waals energy (internal vdw); and ligand intramolecular hydrogen bond energy (internal H‐bond) were considered for calculating the fitness function of GOLD score. The protein–ligand hydrophobic contact was encouraged by making an empirical correction by multiplying external vdw score with 1.375. The fitness function has been optimized for the prediction of ligand binding positions.

Gold score = S (hb_ext) + S (vdw_ext) + S (hb_int) + S (vdw_int),

where S (hb_ext) was the protein–ligand hydrogen bond score, S (vdw_ext) was the protein–ligand van der Waals score, S (hb_int) was the score from intra molecular hydrogen bond in the ligand, S (vdw_int) was the score from intramolecular strain in the ligand.

### 2.18 Statistical analysis

All statistical analyses were conducted using the GraphPad Prism software. Data are expressed as mean ± standard deviation. A nonparametric *t*‐test (Mann–Whitney *U* test) was employed for between‐group comparisons. A *p* < 0.05 was considered statistically significant.

## 3 RESULTS

### 3.1 Characterization of icariin‐loaded PLGA microspheres

The icariin‐loaded PLGA microspheres were synthesized and characterized by Drug Delivery Technology Department, Industrial Technology Research Institute, Hsinchu, Taiwan. The mean particle size of PLGA–icariin in this batch was 10.8 μm. The recovery rate was estimated to 90%. The encapsulation efficiency in this batch was near to 78.2%. And the drug loading efficiency of PLGA–icariin microsphere reached to 21.7% in this batch (Table S2).

### 3.2 PLGA–icariin preserved visual function by inhibiting the RGC apoptosis

**FIGURE 1:**
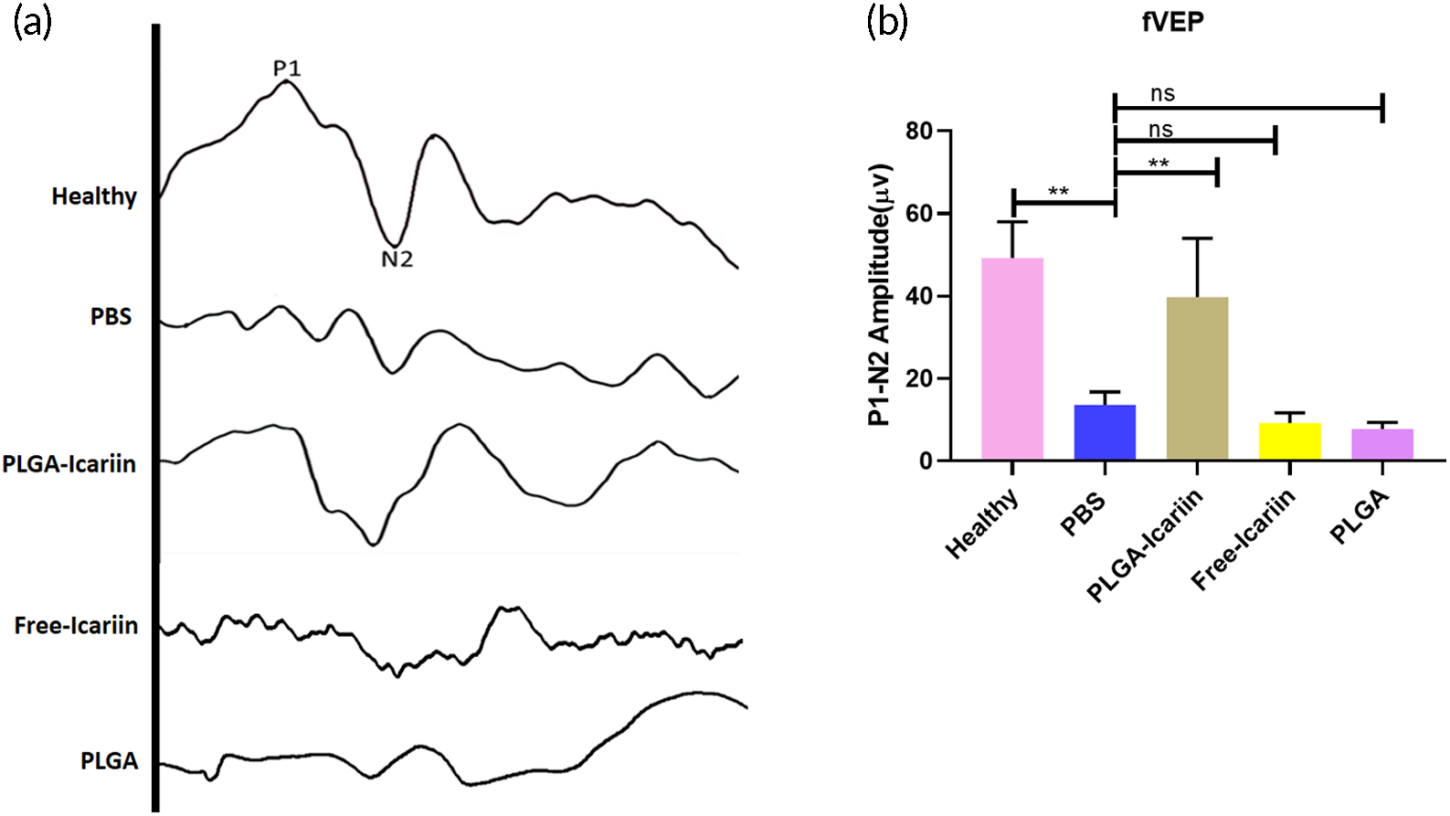
Significant recovery of the functional visual circuitry 28 days after intravitreal injection of PLGA–icariin, as evidenced by the fVEP measurements. (a) Representative fVEP signals with labeled P1‐N2 amplitude for each group measured 28 days after treatment. (b) The P1‐N2 amplitude significantly depreciated in the PBS‐treated, free icariin‐treated, and placebo‐PLGA microsphere‐treated groups but significantly recovered in the PLGA–icariin‐treated group. **p ≤ 0.01, *n* = 6 in each group. fVEP, flash visual evoked potential; PBS, phosphate‐buffered saline; PGLA, poly(lactide‐co‐glycolide)

In the event of rAION, visual circuitry is disturbed because of axonal damage and loss of the RGCs. Four weeks after ON infarct, the P1‐N2 amplitude was calculated to be 49.24 ± 8.78, 17.21 ± 6.24, 39.78 ± 14.23, 8.93 ± 2.47, and 7.65 ± 1.60 μV in the healthy, the PBS‐treated, the PLGA–icariin‐treated, the free icariin‐treated, and the placebo‐PLGA microsphere‐treated groups, respectively (Figure 1). A significant 2.3‐fold increase in the amplitude of the PLGA–icariin‐treated group was observed compared with the PBS‐treated group at Day 28 (*p* = 0.0043). This essentially translates to the significantly preserved visual circuit in the PLGA–icariin‐treated group compared with the PBS‐treated group. Whereas treatment with placebo‐PLGA microspheres or free icariin did not preserve the visual function 28 days after ON infarct.

**FIGURE 2:**
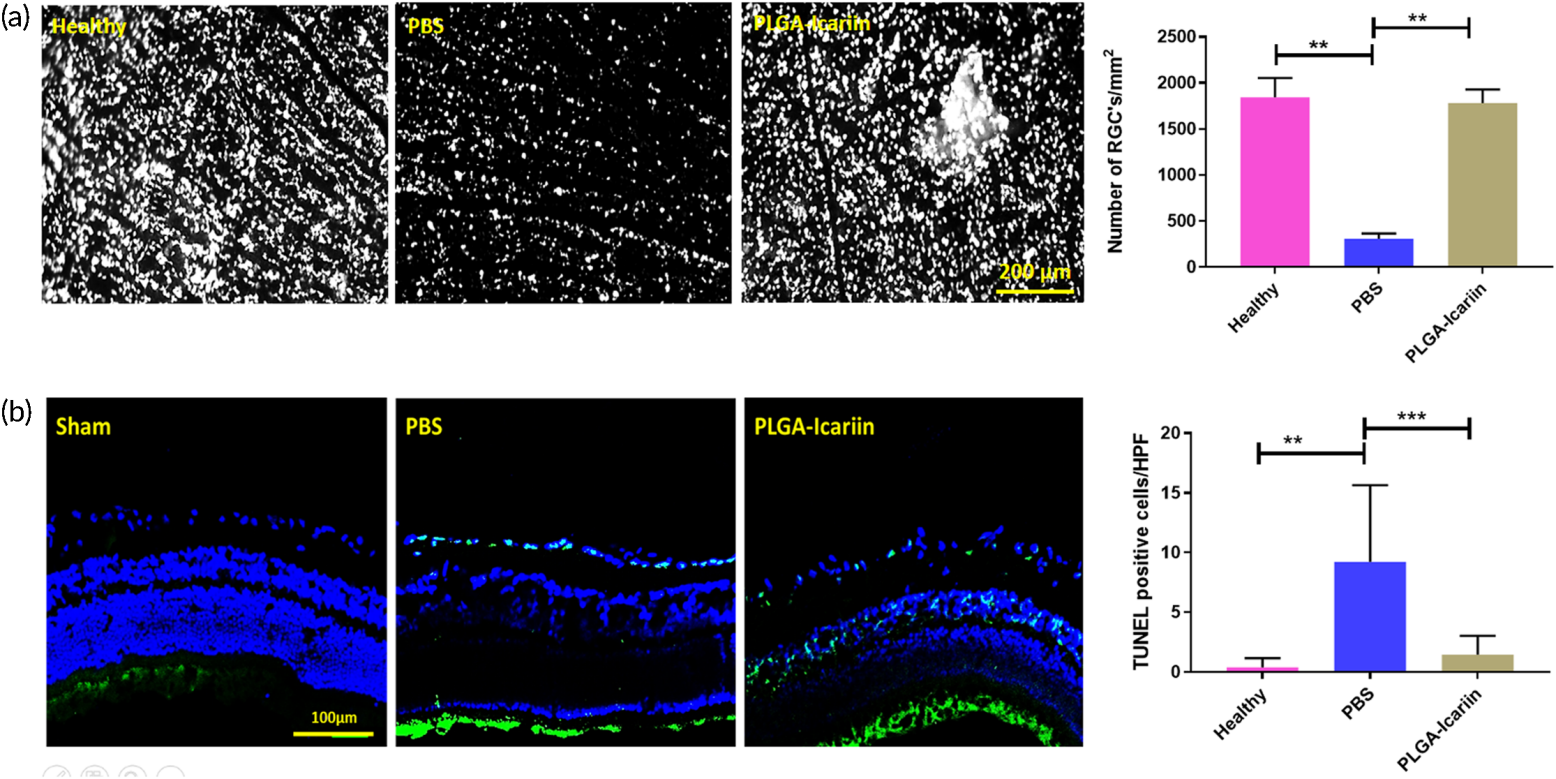
The protective effect of PLGA–icariin on RGCs 28 days after optic nerve infarct. (a) The RGCs (white spots) on the central retina. Graphical representation of the RGC density compared among the different groups. (b) TUNEL‐positive cells (green) in contrast to the other cells (blue) in the retina. Quantification of the TUNEL‐positive cells under high‐power field among each group. ***p ≤ 0.001, ****p ≤ 0.0001, *n* = 6 in each group. PGLA, poly(lactide‐co‐glycolide); RGC, retinal ganglion cell; TUNEL, terminal deoxynucleotidyl transferase dUTP nick end labeling

We measured the density of the RGCs on Day 28 after rAION induction on a retinal flatmount were 1841.667 ± 211.68, 502 ± 432.03, and 1780.43 ± 148.6 cells/mm^2^ in the healthy, the PBS‐treated, and the PLGA–icariin‐treated group, respectively. The RGC density at the central retina in the PBS‐treated group exhibited a significant loss in the RGC population (3.5‐fold) compared with the healthy group, whereas the RGC density in the PLGA–icariin‐treated group was significantly increased (2.17‐fold) compared with the PBS‐treated group (*p* = 0.0095, *p* = 0.0061; Figure 2a). This suggests the potential of PLGA–icariin to rescue damaged RGCs.

To support the above findings, we compared the number of apoptotic RGCs in the RGC layer among the groups using a TUNEL assay on the retinal cryosections. The average numbers of TUNEL‐positive cells in the RGC layer in the healthy, PBS‐treated, and PLGA–icariin‐treated groups were 0.38, 9.21, and 2.05 cells, respectively (Figure 2b). TUNEL‐positive cells were significantly increased in the PBS‐treated group (23.69‐fold) compared with the healthy group and were significantly reduced in the PLGA–icariin‐treated group (4.47‐fold) compared with the PBS‐treated group (*p* ≤ 0.0001, *p* = 0.0006).

### 3.3 Attenuation of ON edema and macrophage infiltration after PLGA–icariin treatment

**FIGURE 3:**
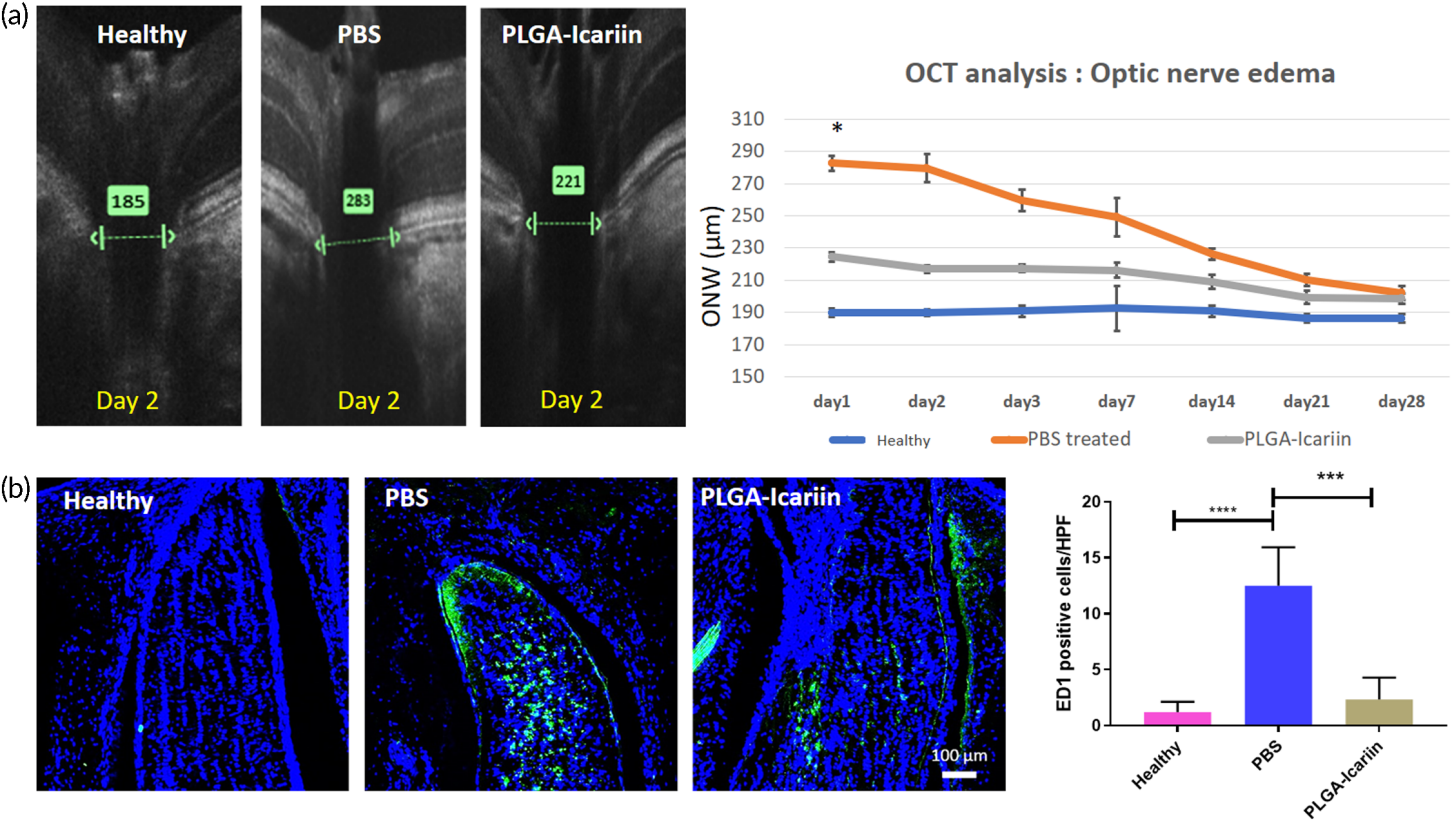
The OCT performed on the ONH reveals that edema of Bruch's membrane resolved earlier in the timeline of the rAION progression along with reduced macrophage infiltration in the ONH. (a) Representative OCT image showing the different layers of the retina at the ONH. The green arrow indicates the distance measured between the same retinal layer on either ends of the ONH. Graphical quantification presents the width of the optic nerve over different time points in all groups. (b) Immunohistochemistry staining for macrophages bearing the ED1 marker (green) signals in contrast to the DAPI (blue) signals in all groups. The graphical quantification of the macrophage density in different groups. *p ≤ 0.05, ***p ≤ 0.001; *n* = 6 in each group. OCT, optical coherence tomography; rAION, rat model of anterior ischemic optic neuropathy

On assessing the damage incurred after rAION via OCT, we found that ONH edema was evident since Day 1. At this point in time, the opening of Bruch's membrane was the widest in the PBS‐treated group, and it gradually decreased with the progression of the disease, resolving after about 7 days. Contrarily, a significant reduction in edema was observed at the ONH in the PLGA–icariin‐treated group on Day 1 (with an approximate average of 224.5 μm) compared with the PBS‐treated group (with an approximate average of 282.6 μm) on Day 1 after infarct (*p* = 0.018), whereas the healthy group served as a baseline (with an approximate average of 185 μm) (Figure 3a). The measurements of ON edema were not significantly different in all groups at 7 days after rAION induction. The high severity of ON edema earlier in the disease progression suggests that the maximum damage is incurred earlier in the timeline. It was found that ON edema can resolve as early as 1 day after PLGA–icariin treatment as evidence of its therapeutic potential.

Four weeks after rAION induction, the number of ED1‐positive macrophages was found to be 1.2 ± 0.7, 12.5 ± 3.4, and 2.28 ± 1.9 cells/HPF in the healthy, PBS‐treated, and PLGA–icariin‐treated groups, respectively. The number of infiltrating macrophages in the PBS‐treated group was 10.4‐fold (*p* ≤ 0.0001) and 5.36‐fold (*p* = 0.0002) higher than those in the healthy group and in the PLGA–icariin‐treated group, respectively (Figure 3b).

### 3.4 The binding of icariin to CEBP‐β induced granulocyte colony‐stimulating factor production

**FIGURE 4:**
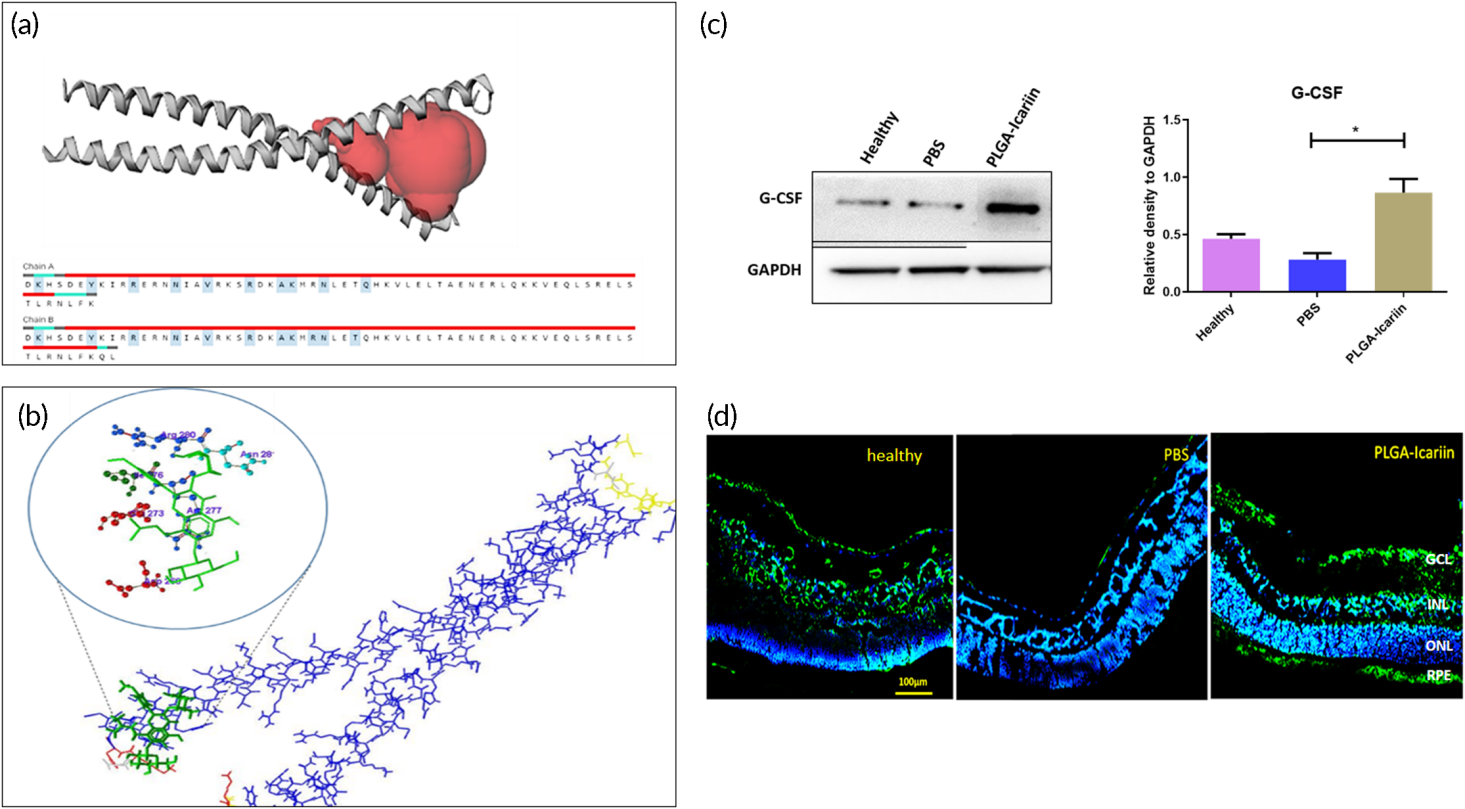
Icariin interacts with CEBP‐β to regulate G‐CSF production in retinal cells. (a) Docking studies using the GOLD software showing the interaction of icariin (green) with CEBP‐β (blue). (b) Active site of CEBP‐β showing the DNA‐binding site for CEBP‐β. (c) Immunoblot analysis for the G‐CSF levels in the retina and quantification for the same. (d) Immunohistochemistry staining for G‐CSF demonstrates its presence in different treatment conditions in the retina. *p ≤ 0.05, *n* = 3 in each group. CEBP‐β, CCAAT enhancer binding protein beta. G‐CSF, granulocyte colony‐stimulating factor

The CEBP‐β is a known regulator of the granulocyte colony‐stimulating factor (G‐CSF) promoter to control G‐CSF expression.^23^, ^24^ The potential interaction between CEBP‐β and icariin was evaluated by using the GOLD bioinformatics software. Structures prepared by obtained the crystal structure from protein data bank (CEBP‐β: 1GTW). For molecular docking, active site of these enzymes was predicted using Castp server. Icariin compound constructed and optimized in Chemsketch software. The chain A of CEBP‐β opted and hydrogen atoms were incorporated into the enzymes for molecular docking studies. Docking of icariin into CEBP‐β active site was performed and the docking evaluations considered based on GoldScore fitness functions. From the molecular docking of Icariin into active site of CEBP‐β, hydrogen bond observed between the hydrogen atom (H4) of Icariin and oxygen atom of ASN281 in CEBP‐β with docking score of 42.77. Icariin was found to interact with CEBP‐β by binding to its leucine zipper at the C‐terminal (Figure 4a,b).

Immunoblot analysis of the retina sample against the G‐CSF antibody also demonstrated that G‐CSF production was decreased by 1.6‐fold (*p* = 0.0286) after rAION induction whereas treatment with PLGA–icariin was able to increase the G‐CSF production by 3.07‐fold (*p* = 0.0286; Figure 4c). In addition, after immunostaining the retinal cross‐section, the expression of G‐CSF was evident throughout the RGC and RPE layers in the PLGA–icariin‐treated group and the healthy group, whereas in the PBS‐treated groups, no expression of G‐CSF was detected in all retinal layers (Figure 4d). Besides, the expression of G‐CSF at RGC and RPE layer was higher in the PLGA–icariin‐treated group compared to the healthy group.

### 3.5 Icariin‐induced G‐CSF triggered noncanonical NF‐κB activation

**FIGURE 5:**
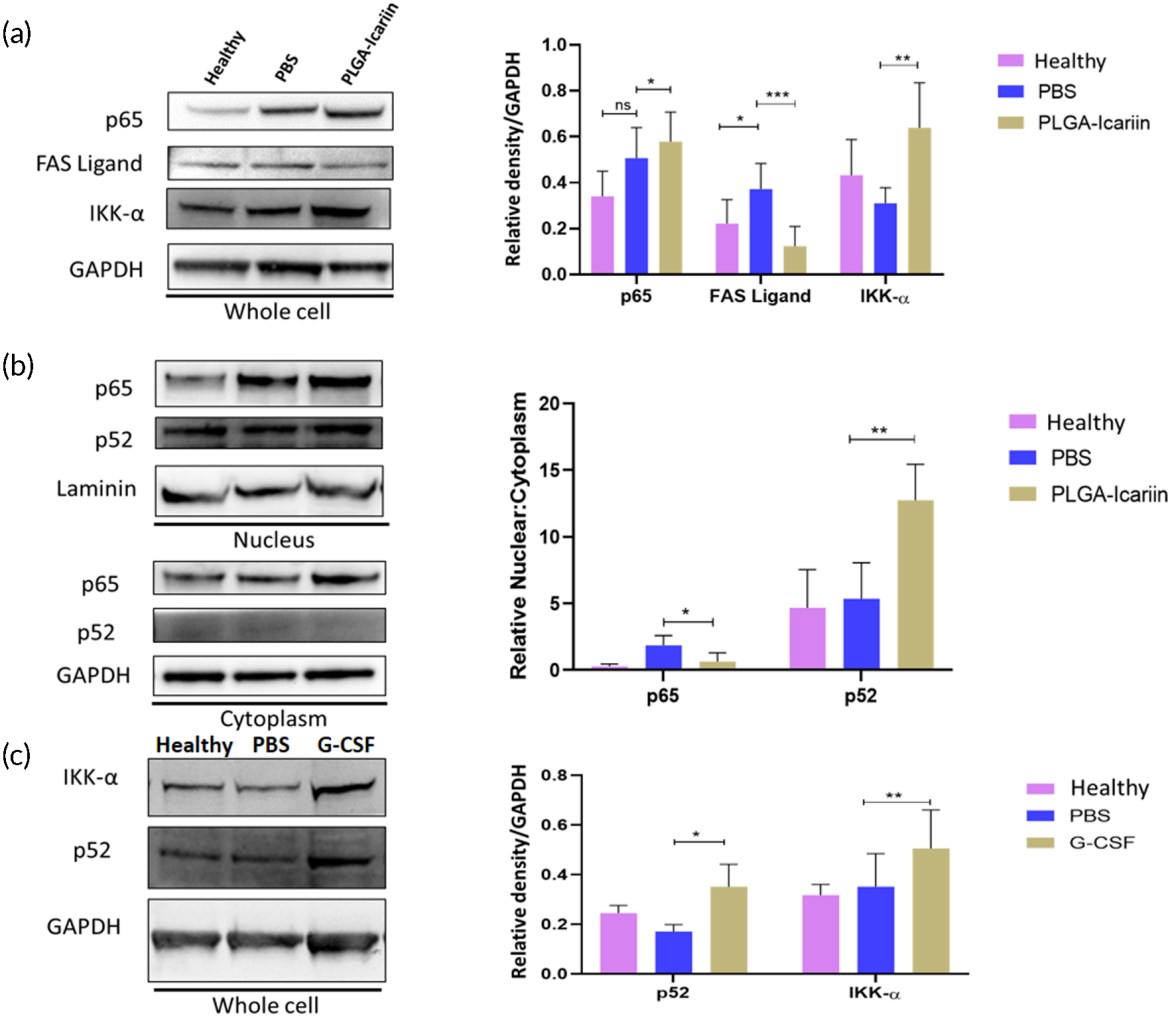
Immunomodulation of NF‐κB to its anti‐inflammatory noncanonical pathway after PLGA–icariin treatment. (a) Western blot analysis of p65, FAS ligand, IKK‐α, and GAPDH. Quantification of these proteins normalized to GAPDH. (b) Immunoblot of p65, p52, and laminin protein levels in the nuclear fraction of the retina. The quantification of these proteins normalized to laminin and Western blot of p65, p52, and GAPDH protein levels in the cytoplasmic fraction of the retina. These protein levels were normalized to GAPDH. (c) Western blot analysis of IKK‐α and p‐52 on the whole retina in the healthy, rAION, and PEG‐G‐CSF groups. ^ns^ *p* > 0.05, *p ≤ 0.05, **p ≤ 0.01, ***p ≤ 0.001, *n* = 3 in each group. G‐CSF, granulocyte colony‐stimulating factor; NF‐κB, nuclear factor kappa B; PGLA, poly(lactide‐co‐glycolide); rAION, rat model of anterior ischemic optic neuropathy

Immunoblot analysis of retina protein samples exhibited an insignificant difference in the NF‐κB (p65) levels between the PLGA–icariin‐treated group and the PBS‐treated group (*p* = 0.4418; Figure 5a). However, FAS ligand, which is downstream of the canonical p65 pathway, was significantly decreased by threefold in the PLGA–icariin‐treated group compared with that in the PBS‐treated group (*p* = 0.0006; Figure 5a). The level of IKK‐α in the PLGA–icariin‐treated group was significantly increased by 2.05‐fold compared to that in the PBS‐treated group (*p* = 0.0043; Figure 5a).

To investigate the possibility of noncanonical NF‐κB activation, the cytoplasmic and nuclear protein were used to determine the ratio of nuclear to cytoplasmic levels of p65 and p52. We found that the ratio of nuclear to cytoplasmic levels of p65 decreased by 2.9‐fold in the PLGA–icariin‐treated group compared with the PBS‐treated group, which confirms that the nuclear localization of NF‐ĸB (p65) was inhibited in the PLGA–icariin‐treated group (*p* = 0.0286; Figure 5b). The ratio of nuclear to cytoplasmic levels of p52 in the PLGA–icariin‐treated group was 2.3‐fold higher than that in the PBS‐treated group (*p* = 0.0022). Taken together, icariin inhibited the level of FAS ligand and induced the level of IKK‐α. Besides, icariin enhanced the p52 nuclear translocation but inhibited the p65 nuclear translocation to induce noncanonical NF‐κB activation.

To examine the role of icariin‐induced G‐CSF in noncanonical NF‐κB activation, we injected the recombinant G‐CSF protein into vitreous cavity to determine the levels of IKK‐α and p52 in rAION. The levels of IKK‐α and p52 were increased by 1.48‐fold and 2.05‐fold, respectively, in the G‐CSF‐treated group compared to those in the PBS‐treated group, respectively (*p* = 0.0400, *p* = 0.0022; Figure 5c).

### 3.6 Icariin‐induced PTEN/PI3K/AKT1 activation via PTEN phosphorylation from IKK‐β

**FIGURE 6:**
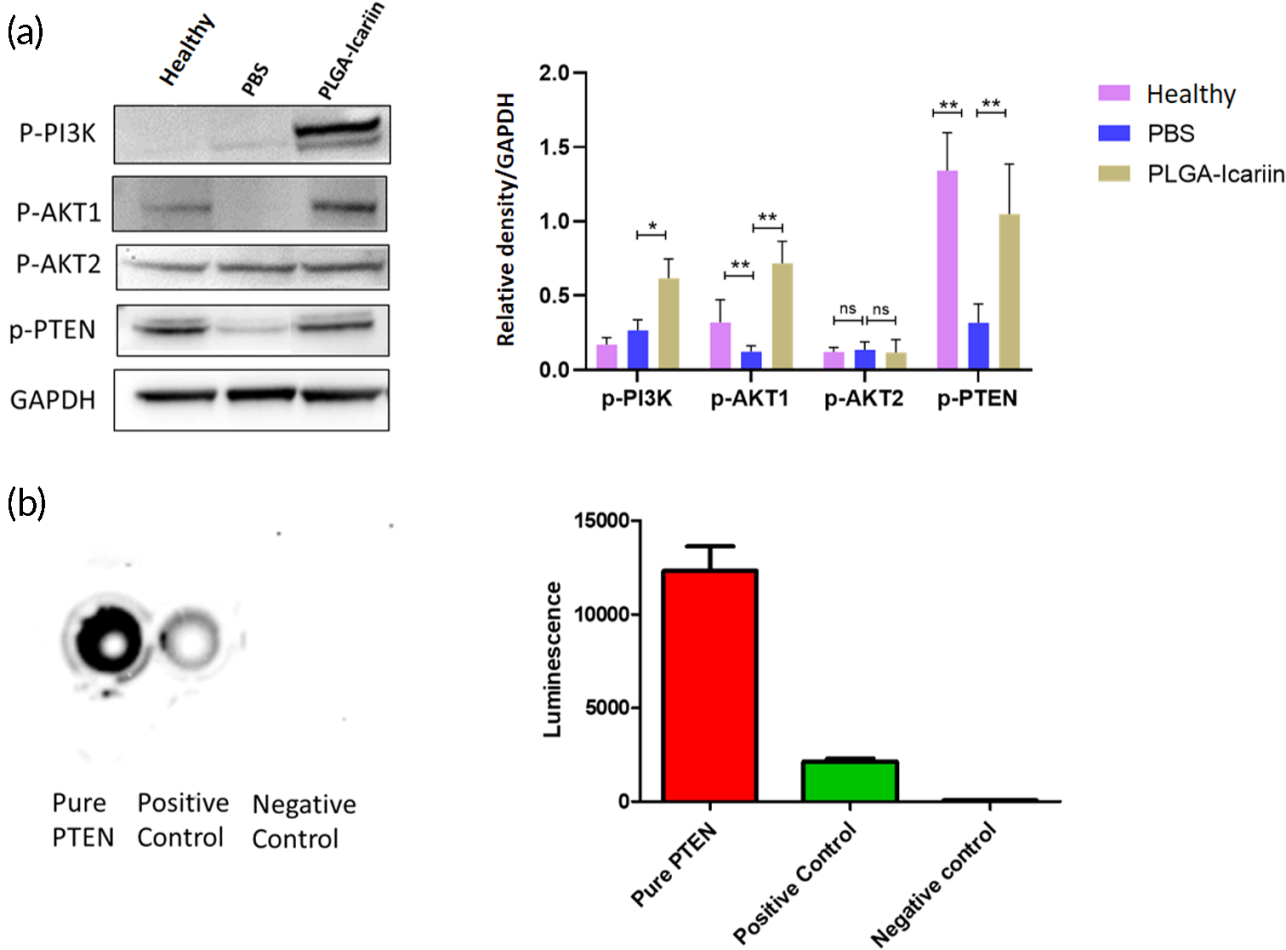
Survival of RGCs mediated by AKT1 and not by AKT2 after PLGA–icariin treatment. Kinase assay for human recombinant IKK‐β against PTEN. (a) Western blot for the protein levels of p‐PI3K, p‐AKT1, p‐AKT2, p‐PTEN, and GAPDH. (b) Quantification of the protein levels normalized to GAPDH. *p ≤ 0.05, **p ≤ 0.01, ^ns^ *p* > 0.05; *n* = 3 in each group. (c) The luminescence after IKK‐β kinase exposure with pure PTEN, positive control, and negative control. (d) Graphical representation of the quantified luminescence signal. **p ≤ 0.01. PGLA, poly(lactide‐co‐glycolide); RGC, retinal ganglion cell

Immunoblot analysis against the phosphorylated PTEN (p‐PTEN) in the retina sample revealed that the level of p‐PTEN in the PLGA–icariin‐treated group was 3.3‐fold higher than that in the PBS‐treated group (*p* = 0.0022; Figure 6a).

The levels of p‐PI3K and p‐AKT1 were significantly increased by 2.32‐fold (*p* = 0.0286) and 5.84‐fold (*p* = 0.0095) in the PLGA–icariin‐treated group, respectively, compared to those in the PBS‐treated group (Figure 6a). However, we did not observe any significant difference of p‐AKT2 level between the PLGA–icariin‐treated group and the PBS‐treated group (*p* = 0.7053).

A kinase assay was performed by incubating recombinant human PTEN along with IKK‐β. The IKKtide or dilution buffer incubated along with IKK‐β served as the positive and negative controls, respectively. A significantly high luminance signal was detected from the PTEN‐incubated chamber compared with the positive control (*p* = 0.0079; Figure 6b). This proves that IKK‐β is a possible kinase for PTEN. To further evaluate its kinase activity, we predicted six serine phosphorylation sites on human PTEN sequence by human IKK‐β using Kine Phos 2.0 at amino acid location 227, 229, 287, 294, 355, and 370 (Figure S1).

### 3.7 Icariin‐induced M2 polarization via JAK1/STAT3 signaling pathway

**FIGURE 7:**
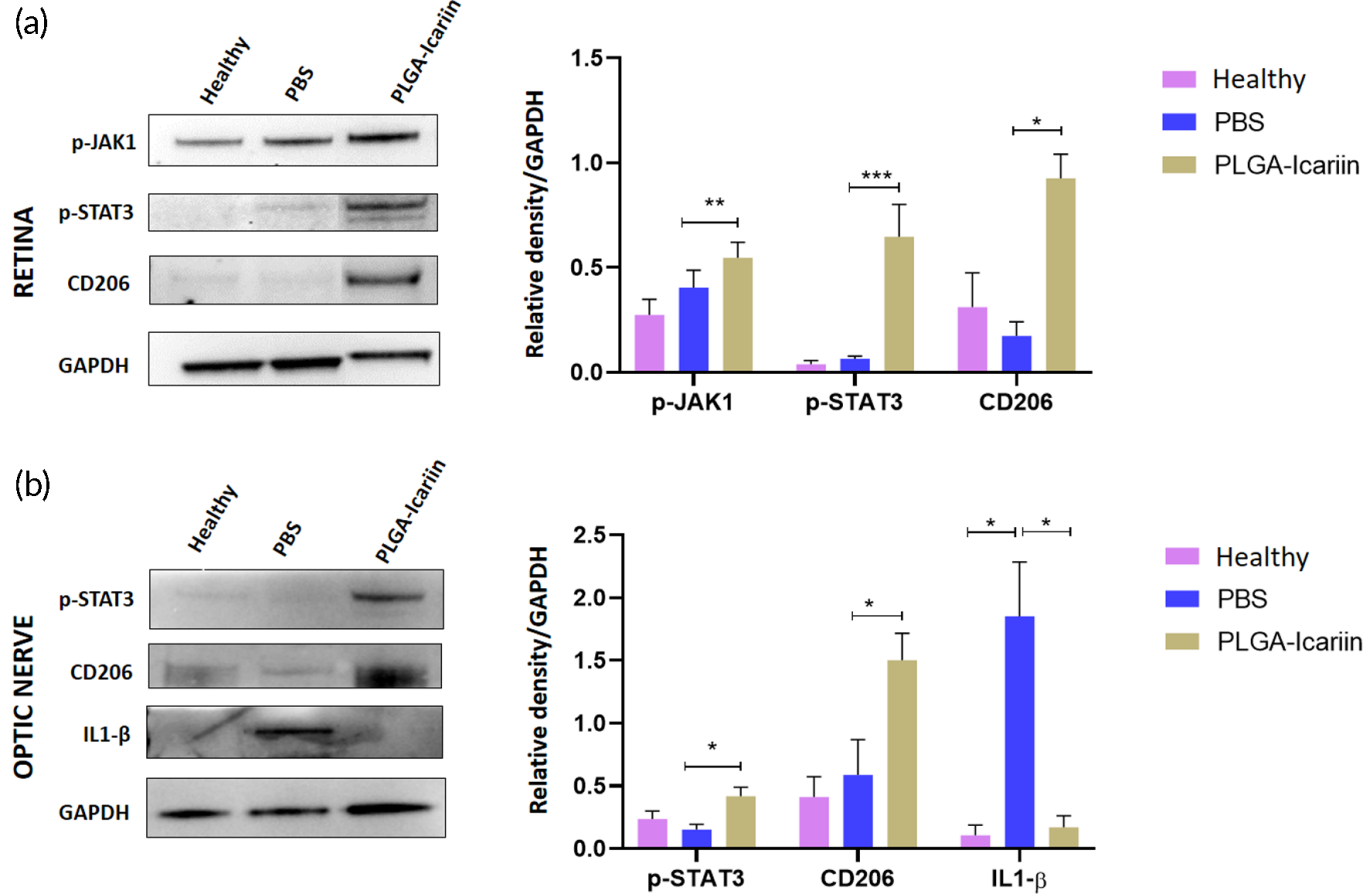
PLGA–icariin promotes STAT3‐mediated M2 macrophage polarization in ON and retina. (a) Western blot analysis of p‐JAK1, p‐STAT3, and CD206 in retina and quantification of the protein levels normalized to GAPDH. (b) Immunoblot of p‐STAT3, CD206, and IL1‐β in ON and quantification of these proteins normalized to GAPDH. *p ≤ 0.05, **p ≤ 0.01, ***p ≤ 0.001; *n* = 3 in each group. ON, optic nerve; PGLA, poly(lactide‐co‐glycolide)

Immunoblot analysis of retinal protein showed that the level of CD206 was increased by 5.31‐fold in the PLGA–icariin‐treated group compared to that in the PBS‐treated group (*p* = 0.0286). The upstream proteins, p‐JAK1 and p‐STAT3, were increased by 1.35‐fold and 9.83‐fold in the PLGA–icariin‐treated group compared to those in the PBS‐treated group, respectively (*p* = 0.0047, *p* = 0.0003; Figure 7a).

Western blotting analysis of the ON sample revealed that the expressions of p‐STAT3 and CD206 were increased by 2.7‐fold and 2.5‐fold, respectively, in the PLGA–icariin‐treated group, compared with the PBS‐treated group (*p* = 0.0286; *p* = 0.0286). These results indicate that PLGA–icariin treatment is capable of inducing STAT3‐mediated M2 macrophage/microglia polarization in the ON and retina. On the contrary, the level of M1 polarization marker, IL‐1β, was significantly decreased by 14.28‐fold in the PLGA–icariin‐treated group compared with the PBS‐treated group (*p* = 0.0159; Figure 7b).

## 4 DISCUSSION

In this study, we demonstrated that treatment with PLGA–icariin preserved the visual function and RGC density by inhibiting RGC apoptosis after ON infarct. In addition, the ON edema and the macrophage infiltration were attenuated by icariin treatment. Moreover, we found that icariin bound the transcriptional factor, CEBP‐β, to induce endogenous G‐CSF production in the retinal cells. The endogenous G‐CSF expression promoted noncanonical NF‐κB activation to further activate PI3K/AKT1 survival pathway and M2 macrophage polarization. Thus, we considered that PLGA microsphere of icariin provides the long‐term neuroprotective effects in the experimental model of ON ischemia via antiapoptotic and anti‐inflammatory actions.

The visual function was protected for 1 month by treatment with PLGA–icariin in the rAION model. Whereas treatment with free icariin or placebo microspheres failed to show any improvement in visual function after ON infarct. This validates the PLGA encapsulation of icariin promotes the extended benefits of icariin (Figure S2). This protective effect on visual function indicated that treatment with PLGA–icariin suppressed RGC apoptosis after the ischemic insult, which was supported by the evidence of RGC density and TUNEL assay. One recent study demonstrated that the NF‐κB mediated apoptosis in fetal rat hippocampal neurons was attenuated by treatment with icariin.^25^ These evidences supported our hypothesis that intravitreal injection of icariin provides neuroprotective effects in the rAION model. Herein, we provided the first evidence that intravitreal injection of icariin inhibited RGC apoptosis and preserved the visual function. However, the protective mechanisms of icariin need further investigation.

The infarct at the ONH causes a breakdown of the BONB, which facilitates the increase in vascular permeability and causes infiltration of the macrophages at the ONH. This further reduces the oncotic pressure gradient, and the hydrostatic pressure in the capillaries of the ON forces out more water, increasing fluid production in the tissue. Thus, the increase in tissue fluid in the ON results in edema at the ON head.^2^, ^3^ In addition, maximum ON edema was observed on Day 1 after infarct, but the resolution of ON edema occurred 1 week after ON infract.^26^ This implies that ON edema is primarily related to damage to the RGCs in the acute stage of ON ischemia. Contrarily, after treatment with PLGA–icariin, ON edema was found to be decreased on Day 1. Therefore, the early relief of swelling pressure on the axons at the ONH may also have contributed to the RGC survival.

After ON infarct, the infiltration of blood‐borne macrophages is the major event of inflammation in the ONH.^27^ BONB stabilization reduces the infiltration of macrophages and the degree of inflammation, hence reduce RGC death in rAION.^28^ The increased ED1‐positive macrophages in the ON of the PBS‐treated group caused increased local inflammation, whereas fewer macrophages observed at the ONH in the PLGA–icariin‐treated group suggest the stabilization of the BONB and posttreatment reduction in macrophage infiltration. This could have also contributed to the resolution of neuroinflammation in the acute stage. Taken together, we suggested that icariin can reduce the breakdown of BONB to attenuate ON edema and macrophage infiltration after ON infarct.

Driven by a binding simulation, we found that icariin has a good binding affinity with CEBP‐β. CEBP‐β is a known regulator of the G‐CSF promoter and is generally present in the system for G‐CSF production.^23^, ^24^ The binding of icariin to the C‐terminal of CEBP‐β, which is known to regulate DNA binding in CEBP‐β,^29^, ^30^ could have increased its DNA‐binding ability and hence promote the increased production of G‐CSF. Moreover, CEBP‐β‐regulated transcription coactivator 2 and 3 (CTRC2/3) inhibit the G‐CSF production, whereas their depletion is followed by increased STAT3 and G‐CSF production via activating the CEBP‐β.^31^ Our findings demonstrated that intravitreal injection of PLGA–icariin highly induced G‐CSF expression in the ganglion cell layer and the retinal pigmented epithelium layer. In addition, our in vitro experiment proved the dose–response relationship between icariin and G‐CSF in the human RPE cell line (Figure S3). We suggested that Icariin could block the binding of CTRC2/3 to enhance G‐CSF production in the retinal cells. G‐CSF is a potent neuroprotective agent through anti‐inflammation and antiapoptosis in the experimental model of ON ischemia.^5^ Thus, we considered that icariin triggers endogenous G‐CSF expression to modulate the neuroinflammation after ON infarct.

NAION is an inflammatory disease similar to many other neurodegenerative diseases.^32^, ^33^, ^34^ Hence, we expected increased levels of NF‐κB (p65) in PBS‐treated group but not in PLGA–icariin‐treated group. But to our surprise, the levels of NF‐κB remained high after treatment with PLGA–icariin. To gain resolution on this situation, we checked the protein levels of FAS ligand in all the groups. FAS ligand is known to upregulate under the canonical NF‐κB (p65) condition but downregulates while in the noncanonical progression of NF‐κB (p52).^35^ FAS ligand was found to be upregulated in the PBS‐treated group while downregulated after PLGA–icariin treatment. This suggested the possibility that although NF‐κB (p65) may have been upregulated in the PLGA–icariin‐treated group, it may not have translocated to the nucleus for the transcription of its downstream products. These doubts were confirmed by the downregulation of the protein levels of p65 in the nucleus in reference to its levels in the cytoplasm. On accessing the p52 levels in the retina, increased translocation of p52 to the nucleus with respect to the cytoplasm was observed in the PLGA–icariin‐treated group compared with the PBS‐treated group, which corresponds to noncanonical NF‐κB progression in the PLGA–icariin‐treated group. The upregulation of the noncanonical NF‐κB pathway was further confirmed by the increase in the IKK‐α levels observed after PLGA–icariin treatment compared with the PBS‐treated group.^22^ Hence, we considered that PLGA–icariin treatment could promote a switch from the canonical to noncanonical NF‐κB pathway. A similar trend was observed after PEG‐G‐CSF treatment, in which an increase in IKK‐α and p52 levels was noted compared to the PBS‐treated group. This demonstrates that PLGA–icariin treatment could promote noncanonical NF‐κB progression via the G‐CSF‐mediated pathway. The noncanonical NF‐κB pathway is antiapoptotic in nature and has been reported to regulate inflammation.^22^ It also provides anti‐inflammatory benefits, whereas its deregulation could promote inflammation.^36^ Although the benefits of noncanonical NF‐κB are overwhelming, little is known about the mechanism that regulates its switch.^37^

With the aim of investigating the interactions that favor the switch to the noncanonical NF‐κB pathway, we observed that the upregulated p65 was unable to translocate to the nucleus after PLGA–icariin treatment, which could be explained by the inability of IKK‐β to phosphorylate p65.^38^ We also observed that PTEN is phosphorylated rather than being downregulated. The PI3K/AKT pathway is favored by downregulated PTEN, which otherwise gets phosphorylated by scavenging the phosphates from PI3K and inhibits the phosphorylation of PIP2, hence inhibiting the PI3K/AKT pathway.^39^ Therefore, PTEN phosphorylation and PI3K/AKT progression are generally mutually exclusive, indicating that another kinase must have acted on the PTEN instead. A previous study demonstrated the ability of the NF‐κB pathway to modulate the PTEN activity via the observation of decreased PTEN levels in IKK‐β(+,+) cells but not in the IKK‐β(−,−) cells.^40^ There have also been reports suggesting that the NF‐κB pathway has an inhibitory effect on the PTEN in the form of a positive feedback loop.^41^, ^42^ Based on the observations and arguments stated above, we suspected the possibility of IKK‐β's alternative role in the phosphorylation of PTEN. On assessing the situation via kinase assay, we confirmed that IKK‐β has the potential for PTEN phosphorylation. In addition, in silico evidence also indicated the ability of human IKK‐β to phosphorylate the human PTEN sequence. This explains the unavailability of IKK‐β for p65 phosphorylation as well as the presence of the upregulated p‐PTEN in the presence of the upregulated PI3K/AKT pathway. This finding suggests that the phosphorylation target of IKK‐β plays a decisive role in the progression of the NF‐κB pathway. The phosphorylation of p65 by IKK‐β leads to the canonical whereas the phosphorylation of PTEN by IKK‐β to the noncanonical progression of NF‐κB. There is also a possibility that the phosphorylation of PTEN is not the sole alternative target for IKK‐β but is rather an important target, as the noncanonical NF‐κB pathway also requires the upregulated PI3K/AKT pathway for its functioning.^43^ NF‐κB (p65) progression via the canonical pathway increases inflammation in rAION and promotes neurodegeneration. Conversely, its switch to the noncanonical pathway after PLGA–icariin treatment, owing to its anti‐inflammatory nature, is desired over the canonical progression.^36^ The mechanism of this switch via the exhaustion of the availability of IKK‐β for phosphorylation of p65 could provide a new approach for targeting inflammation.

RGC survival along with G‐CSF upregulation suggested that G‐CSF influenced the underlying mechanism. The PI3K/AKT survival pathway mediated by the upregulated G‐CSF elucidated the rescue of the RGCs along with upregulated PTEN phosphorylation in the retina. Here, we report that the upregulation of the PI3K/AKT survival pathway via AKT1 upregulation is sufficient to bring about considerable RGC survival, whereas AKT2 has no role in this process, which is consistent with the findings of a previous study.^24^

Macrophages assume either M1 or M2 polarization and previous studies have proved that IKK‐α activation inhibits M1 phenotype of the macrophages^44^ whereas inactivation of IKK‐α is known to increase inflammation in mice,^45^ which suggested the noncanonical NF‐κB signaling pathway could regulate the macrophage polarization. When analyzed by the C206 marker, increased M2 polarized macrophages were recorded at the ON head after treatment with PLGA–icariin. While increased M2 macrophage polarization was found to be promoted via the upregulation of phosphorylated STAT3 (p‐STAT3).^46^, ^47^ p‐STAT3 upregulation is downstream of JAK activation, which is known to be activated upon G‐CSF–G‐CSF receptor activation.^48^ Owing to their high phagocytic ability and ability to produce Type 1 inflammatory cytokines, such as IL‐1β and tumor necrosis factor‐α, M1‐type macrophages are inflammatory in nature.^49^, ^50^ As M2 polarization is anti‐inflammatory in nature, its benefits were analyzed at the site of primary inflammation, the ON head.^5^ Increased IL‐1β after rAION represents an upregulated inflammatory response, whereas decreased IL‐1β expression after treatment with PLGA–icariin indicates its anti‐inflammatory effects.^5^, ^51^ We also observed the M2 macrophage/microglia polarization in the retina after PLGA–icariin treatment. As the canonical NF‐κB progression drives M1 macrophage polarization^52^; based on our observation, the noncanonical NF‐κB progression regulates M2 macrophage/microglia polarization and contributes to the anti‐inflammatory phenomenon exerted by M2 polarized macrophages/microglia. The degree to which the progression of NF‐κB regulates macrophage polarization requires further study.

## 5 CONCLUSION

**FIGURE 8:**
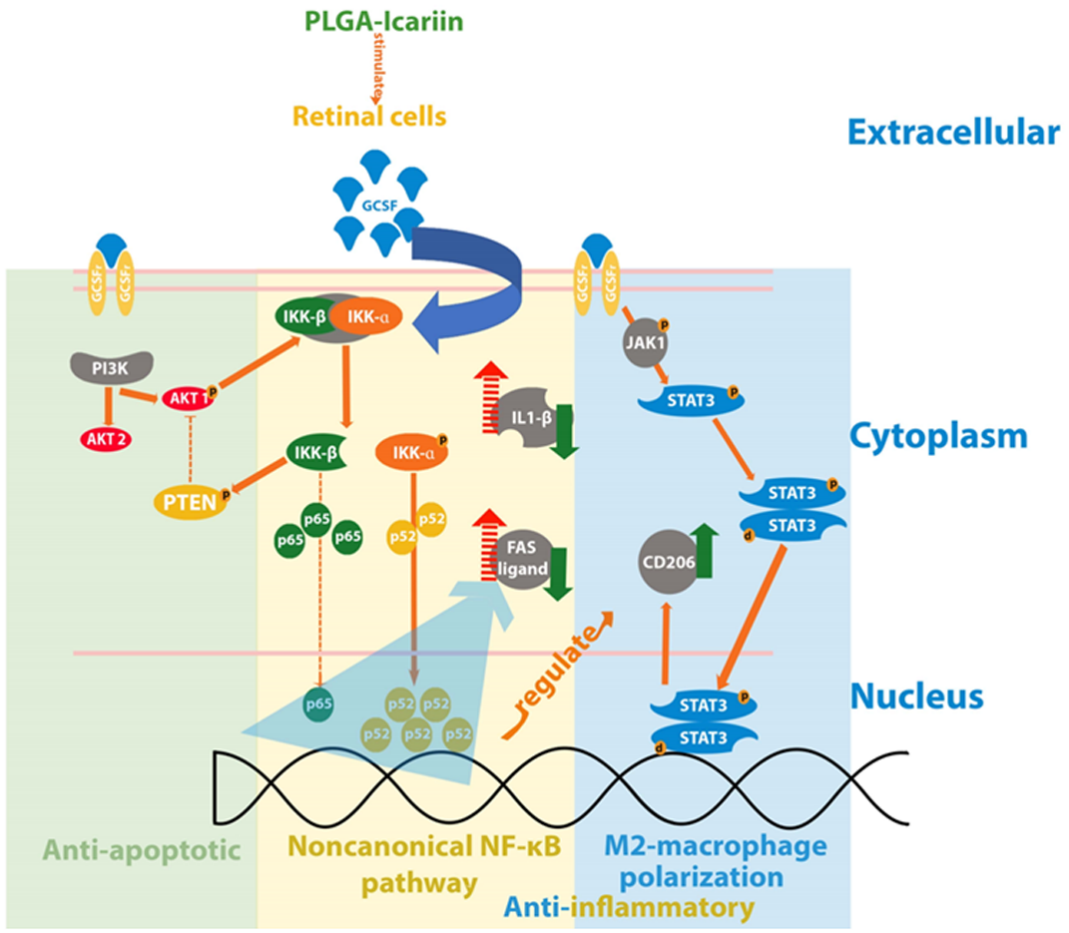
The neuroprotective potential of PLGA–icariin treatment summarized by the survival of RGCs, anti‐inflammatory activity, and M2 macrophage polarization along with their interactions. PGLA, poly(lactide‐co‐glycolide); RGC, retinal ganglion cell

To summarize, our findings demonstrated the long‐term neuroprotective potential of a single dose of PLGA–icariin against ON ischemia. Icariin‐induced G‐CSF leads to noncanonical NF‐κB activation which results in antiapoptotic and anti‐inflammatory benefits to prevent the loss of visual function and RGCs after ON infarct (Figure 8). Based on our results, we believed that intravitreal injection with PLGA microsphere of icariin is an ideal drug delivery system to treat the progressive and long‐lasting RGC death and vision loss in patient with ON ischemia.

## CONFLICT OF INTERESTS

The authors declare no conflicts of interest.

## AUTHOR CONTRIBUTIONS

Rong‐Kung Tsai, Yao‐Tseng Wen, and Tushar Dnyaneshwar Desai conceptualized and designed the research project. Rong‐Kung Tsai, Felice Cheng, Chia‐Ching Chen, Chien‐Lin Pan, Keh‐Liang Lin, and Yao‐Tseng Wen contributed to all the necessary materials, financial support, and laboratory facilities. Tushar Dnyaneshwar Desai and Yao‐Tseng Wen executed all the experiments while Jayasimha Rayalu Daddam performed the in silico analysis in this study. Tushar Dnyaneshwar Desai and Yao‐Tseng Wen contributed to the discussion and interpretation of the raw data. Yao‐Tseng Wen and Tushar Dnyaneshwar Desai contributed to data evaluation and drafting. All authors read and approved the final manuscript.

### PEER REVIEW

The peer review history for this article is available at https://publons.com/publon/10.1002/btm2.10289.

## Supporting information


**Appendix** S1: Supporting information

## Data Availability

The data supporting the finding of this study are available within the article and its Supplementary Information files or available from the corresponding author on reasonable request. This study includes no data deposited in external repositories.
