# Supplementary material for: Long term therapeutic effects of icariin‐loaded PLGA microspheres in an experimental model of optic nerve ischemia via modulation of CEBP‐β/G‐CSF/noncanonical NF‐κB axis
Source: Bioeng Transl Med. 2022 Jan 7;7(2):e10289. doi: 10.1002/btm2.10289 (PMC9115698; doi:10.1002/btm2.10289)
Supplement: Supplementary file 1 — Appendix S1: Supporting information [file BTM2-7-e10289-s001.pdf]

Supplementary Materials for

Long term therapeutic effects of icariin-loaded PLGA microspheres in an experimental model of optic nerve ischemia via modulation of CEBP-β/G-CSF/non-canonical NF-kB axis

Yao-Tseng Wen[1], Tushar Dnyaneshwar Desai[1], Felice Cheng[3], Chia-Ching Chen[3], Chien-Lin Pan[3], Jayasimha Rayalu Daddam[4], Keh-Liang Lin [5], Rong-Kung Tsai[1,2]Correspondence to:
rktsai@tzuchi.com.tw

This PDF file includes:

Table S1 to S3 and Figure S1 to S4

Table S1. Pharmacokinetic Parameters of free icariin and PLGA-icariin in the vitreous humor

Parameter	Units	Free icariin	PLGA-icariin
AUC_{0-last}	ng*d/mL	11.691 ± 0.269	38,711.13± 1,836
$t_{1/2}$	d	0.06± 0.01	23.6 ± 7.2
C_{max}	ng/mL	1194.86 ± 27.24	4,387.35± 57.24
T_{max}	d	0.01	12
C_{last}	ng/mL	0.00107 ± 0.00084	0.00161 ± 0.00044
T_{last}	d	1	55

Table S2. characteristics of icariin-loaded PLGA microspheres

Sample	Mean Particle size (μm)	Recovery rate (%)	Encapsulation efficiency (EE) (%)	Drug loading efficiency (LE) (%)
PLGA-icariin	10.8 (d90：18.9)	90.0	78.2	21.7

Recovery rate=recovered mass/ (initial drug + polymer)
EE=recovered drug mass/ initial drug mass
LE=drug percentage in the final sample

Table S3: Icariin cannot pass the blood–retina barrier. The characteristics of icariin were studied based on Lipinski's rule of five.

Ligand name	MILogP	TPSA	N ATOM	nON	nOHNH	N VIOLA-TIONS	nROTB	VOLUME
Icariin	1.67	238.21	48	15	8	3	9	589.92

Summary Result				
Protein Name	Predicted Phosphorylated Sites			
	Serine(S)	Threonine(T)	Tyrosine(Y)	Histidine(H)
P60484	6	0	0	0

P60484

Locations (AA)	Phosphorylated Sites	SVM score	Catalytic Kinases	Predictive Models	
				SVMs	Logo
227	KIYSSNSGP	0.683143	IKK	SVM	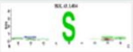
229	YSSNSGPTR	0.525708	IKK	SVM	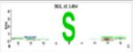
287	PEETSEKVE	0.511406	IKK	SVM	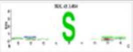
294	VENGSLCDQ	0.66591	IKK	SVM	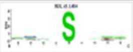
355	VEEPSNPEA	0.58852	IKK	SVM	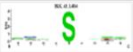
370	TPDVSDNEP	0.658757	IKK	SVM	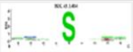

```
MTAIIKEIVS RNKRRYQEDG FDLDLTYIYP NIIAMGFPAE RLEGVYRNNI DDVVRFLDSK 60

HKNHYKIYNL CAERHYDTAK FNCRVAQYPF EDHNPPQLEL IKPFCEDLDQ WLSEDDNHVA 120

AIHCKAGKGR TGVMICAYLL HRGKFLKAQE ALDFYGEVRT RDKKGVTIPS QRRYVYYYSY 180

LLKNHLDYRP VALLFHKMMF ETIPMFSGGT CNPQFVVCQL KVKIYSSNSG PTRREDKFMY 240
---------- ---------- ---------- ---------- ------S--- ---------- IKK
---------- ---------- ---------- ---------- --------S- ---------- IKK

FEFPQPLPVC GDIKVEFFHK QNKMLKKDKM FHFWVNTFFI PGPEETSEKV ENGSLCDQEI 300
---------- ---------- ---------- ---------- ------S--- ---------- IKK
---------- ---------- ---------- ---------- ---------- ---S------ IKK

DSICSIERAD NDKEYLVLTL TKNDLDKANK DKANRYFSPN FKVKLYFTKT VEEPSNPEAS 360
---------- ---------- ---------- ---------- ---------- ----S----- IKK

SSTSVTPDVS DNEPDHYRYS DTTDSDPENE PFDEDQHTQI TKV 403
---------S ---------- ---------- ---------- --- IKK
```

Figure S1: IKK-β is a potential kinase for PTEN.

Phosphorylation sites on PTEN by IKK-β predicted using the Kine Phos 2.0 prediction bioinformatics tool.

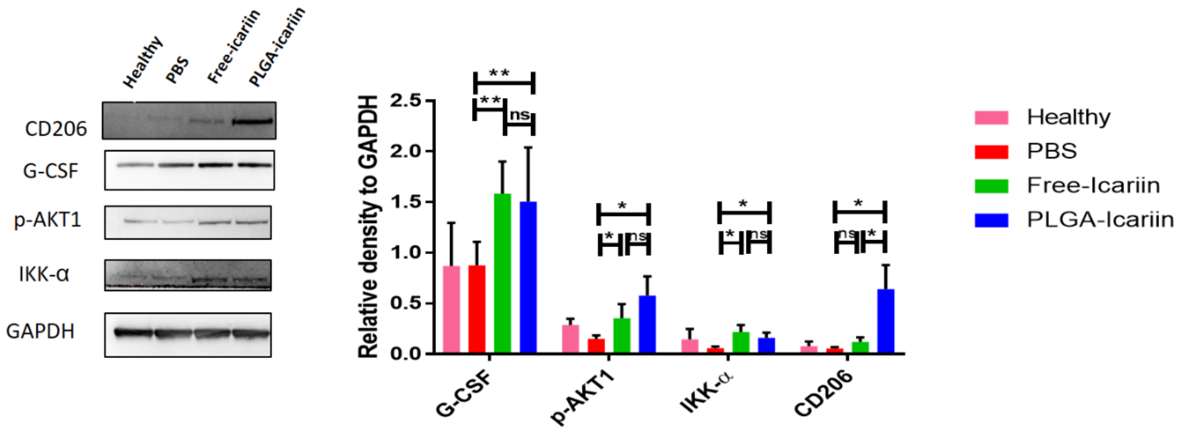

Figure S2. Many signaling pathway was evaluated in the healthy, the PBS-treated, free-icariin-treated, and PLGA-icariin-treated group by Western blotting analysis.

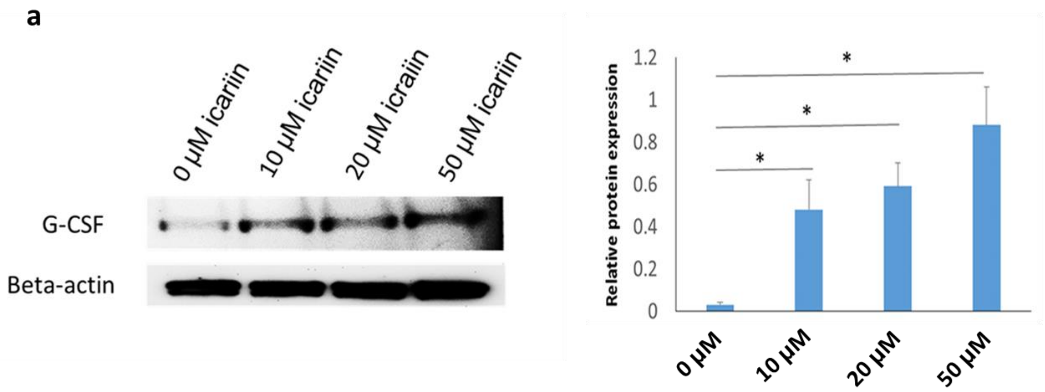

Figure S3. Icariin stimulates the G-CSF progression in dose dependent manner.

Immunoblot analysis for G-CSF expression of ARPE19 cells exposed to different doses of icariin

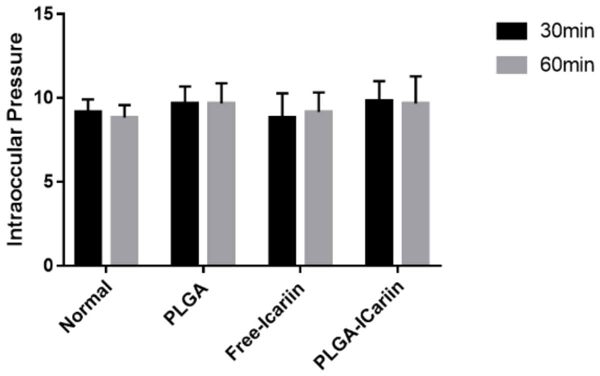

Figure S4. The IOP measurement 30 min and 60 min after intravitreal injection of placebo PLGA microspheres, free icariin, and icariin-loaded PLGA microspheres
